# Open-Label Clinical Trial on the Impact of Autologous Dendritic Cell Therapy on Albuminuria and Inflammatory Biomarkers (Interleukin-6, Interleukin-10, Tumor Necrosis Factor α) in Diabetic Kidney Disease (DKD)

**DOI:** 10.3390/cimb46120816

**Published:** 2024-12-02

**Authors:** Jonny, Enda Cindylosa Sitepu, Bhimo Aji Hernowo, Linda Chiuman, I Nyoman Ehrich Lister, Terawan Agus Putranto

**Affiliations:** 1 Faculty of Medicine, Dentistry, and Health Science, Universitas Prima Indonesia, Medan 20118, Indonesia; lindachiuman@unprimdn.ac.id (L.C.); nyoman@unprimdn.ac.id (I.N.E.L.); terawanagusputranto@unprimdn.ac.id (T.A.P.); 2 Faculty of Medicine, Universitas Pembangunan Nasional “Veteran” Jakarta, Jakarta 12450, Indonesia; 3 Faculty of Military Medicine, Indonesia Defence University, Bogor 16810, Indonesia; 4 Nephrology Division, Department of Internal Medicine, Gatot Soebroto Central Army Hospital, Jakarta 10410, Indonesia; 5 Indonesia Army Cellcure Center, Gatot Soebroto Central Army Hospital, Jakarta 10410, Indonesia; endacsitepu@gmail.com (E.C.S.); hernowobhimo@gmail.com (B.A.H.)

**Keywords:** diabetic kidney disease, dendritic cells, albuminuria, Tumor Necrosis Factor-alpha, Interleukin-10, Interleukin-6, Inflammation, clinical trial

## Abstract

The prevalence of type 2 diabetes mellitus (T2DM) is increasing worldwide, leading to a higher incidence of diabetic kidney disease (DKD), a major risk factor for end-stage kidney disease (ESKD). This study investigates the effects of autologous dendritic cell (DC) therapy on albuminuria and inflammatory biomarkers (IL-6, IL-10, and TNF-α) in DKD patients. An open-label clinical trial was conducted with 69 DKD outpatients at the Gatot Soebroto Army Central Hospital (RSPAD GS). Each subject received a single DC injection, with evaluations of urinary albumin-creatinine ratio (UACR) and inflammatory biomarkers at baseline and 4 weeks post-intervention. UACR was measured weekly, while eGFR, IL-6, IL-10, and TNF-α levels were assessed at baseline and week 4. Results indicated a significant reduction in median UACR from 250 mg/g at baseline to 153 mg/g in week 1, with sustained lower levels over 4 weeks (*p* < 0.05). No significant change of eGFR was found (*p* = 0.478). TNF-α levels also significantly decreased from 2.16 pg/mL to 1.92 pg/mL (*p* = 0.03), while IL-6 (*p* = 0.83) and IL-10 (*p* = 0.11) showed no significant change. The reduction in UACR and TNF-α suggests that DC therapy may alleviate albuminuria through anti-inflammatory mechanisms primarily suppressing TNF-α. No significant change in IL-10 levels implies that the anti-inflammatory effect is not mediated by IL-10 enhancement. This study demonstrates the potential of DC therapy as adjunct therapy to reduce albuminuria in DKD patients, with further research needed to explore long-term efficacy, long-term safety, and dosing strategies.

## 1. Introduction

The prevalence of type 2 diabetes mellitus (T2DM) continues to rise, with more than 460 million individuals affected worldwide in 2019, and this number is projected to reach 783 million by 2045 [1,2]. In Indonesia, the prevalence of T2DM has also increased from 1.5% in 2013 to 2.0% in 2018, while the prevalence of individuals with blood glucose testing rose from 6.9% to 8.5% [3]. Currently, Indonesia ranks fourth in the world for T2DM prevalence.

Diabetic kidney disease (DKD) is a serious complication occurring in 20–50% of T2DM patients and is a major risk factor for the development of end-stage kidney disease (ESKD) [4]. DKD is characterized by persistent albuminuria, progressive decline in kidney function, and structural changes in the kidneys such as mesangial expansion and glomerulosclerosis [5]. Each year, there are approximately 2.6 million new cases of DKD globally, and this number is expected to continue to rise [6]. DKD can also occur without albuminuria, known as non-albuminuric DKD, and is often associated with macroangiopathy, interstitial fibrosis, and vascular lesions [7,8,9].

The main therapy for reducing albuminuria in DKD is the use of renin-angiotensin system inhibitors (ACE-I and ARB). The use of mineralocorticoid receptor antagonists (MRAs), such as eplerenone and spironolactone, is also applied, although they carry a risk of hyperkalemia. SGLT2 inhibitors have also shown potential in reducing albuminuria when combined with ACE-I, but the results are often not entirely satisfactory, indicating the need for further research into more effective alternative therapies [10,11].

Autologous dendritic cell (DC) transfer has been widely used for cancer, chronic infections, and autoimmune diseases, but its application in metabolic and degenerative diseases, such as diabetic kidney disease (DKD), remains unexplored [12]. Current research on cell therapy for DKD focuses on using stem cells. While animal studies indicate that stem cell therapy can improve kidney function in DKD, human trials, such as those using allogeneic mesenchymal precursor cells, have yielded limited success [13,14]. Despite the potential of stem cells in metabolic disease therapy, challenges such as reduced efficacy due to metabolic memory, increased cell senescence, and teratoma risk persist [15]. The current evidence supports the immunomodulatory and anti-inflammatory roles of stem cells in DKD treatment; this suggests that other cell-based products with similar mechanisms could also be viable therapeutic options [14].

Dendritic cell (DC) therapy has significant anti-inflammatory effects, as inflammation is one of the main mechanisms causing albuminuria in DKD [16]. Therefore, intervention with DC therapy may suppress inflammation and, in turn, reduce proteinuria in DKD patients. This study aims to demonstrate that DC therapy can suppress inflammation, as indicated by changes in inflammatory biomarkers (IL6, IL-10, and TNF-α), thereby reducing albuminuria in DKD patients.

## 2. Materials and Methods

### 2.1. Study Design

This study is an open-label clinical trial without randomization of subjects and without blinding of the research team. The study design is a quasi-experimental pre-test and post-test. The research procedures were conducted following applicable guidelines and regulations. This study was approved by the Ethics Committee of Gatot Soebroto Army Central Hospital (RSPAD GS) with Ethical Approval No. 72/V/KEPK/2024. All subjects provided written informed consent.

### 2.2. Study Subjects

The study sample consisted of DKD outpatients at the internal medicine clinic of RSPAD GS from April to May 2024. Samples were selected using non-probability consecutive sampling (quota sampling). A minimum of 68 subjects was required to detect significant changes in UACR. Sample size analysis is described in detail in Supplementary Materials S1. Subjects were recruited based on inclusion criteria: (1) male or female over 18 years old, (2) understanding and agreeing to comply with the study procedures with written consent, (3) assessed by the researcher as able to comply with the procedures, (4) judged to have overall good physical and mental health, (5) meeting the diagnostic criteria for Type 2 Diabetes Mellitus (T2DM) as per PERKENI 2021 [17], (6) eGFR ≥ 30 mL/min/1.73 m^2^, (7) Urinary albumin-creatinine ratio (UACR) ≥ 30 mg/g. Patients who met any of the exclusion criteria were not included in the study. The exclusion criteria for this study are: (1) Receiving immunosuppressive treatment; (2) Presence of other kidney diseases; (3) Presence of other diseases that can cause albuminuria; (4) Presence of other types of diabetes (Type 1 diabetes, gestational diabetes, and other types of diabetes); (5) Positive pregnancy test; (6) Presence of immunodeficiency diseases such as human immunodeficiency virus (HIV), hepatitis C virus (HCV), or hepatitis B virus (HBV), (7) Having a condition that requires oxygen supplementation; (8) Diagnosed with invasive cancer and receiving anti-cancer therapy other than hormonal therapy for breast and prostate cancer; (9) History of thromboembolism or genetic predisposition to thromboembolism, or currently undergoing therapy with anti-thromboembolic agents other than low-dose aspirin, (10) Physical or mental disabilities that prevent normal daily activities, (11) In the researcher’s assessment, the presence of medical conditions that may hinder subject participation, (12) Severe obesity (BMI > 40 kg/m^2^) and uncontrolled hypertension: systolic > 180 mmHg/diastolic > 100 mmHg.

Due to ethical considerations, we did not limit routine medication use, including those known to affect UACR, such as RAS inhibitors, GLP-1 Receptor Agonists, and SGLT-2 inhibitors. However, all concomitant drugs used during the study were recorded.

### 2.3. Study Procedure

Each subject underwent a 5-week clinical trial, including a screening phase, baseline laboratory tests, blood sampling for DC preparation, DC injection, weekly UACR evaluations for 4 weeks post-injection, and a final laboratory evaluation 4 weeks after DC injection. eGFR was measured at baseline and 4 weeks after DC injection. Inflammatory biomarker assessments (IL-6, IL-10, and TNF-α) were performed at baseline and 4 weeks after DC injection. The research protocol was approved by the Health Research Ethics Committee of RSPAD GS.

### 2.4. DC Preparation

Forty cc of peripheral blood was collected. Peripheral Blood Mononuclear Cells (PBMCs) were isolated and incubated with GM-CSF (Granulocyte Macrophage Colony Stimulating Factor) and IL-4 (Interleukin-4) for 5 days to form dendritic cells. Antigen was incubated for 2 days to induce DC maturation. The cell products were injected subcutaneously into the upper arm. The exact number of autologous dendritic cells administered varies among patients, depending on individual yields. No adjustment is made to standardize the cell count across patients. The entire product, derived from 40 cc of peripheral blood, is infused, resulting in a dose that ranges between 0.5 and 8 million DCs.

### 2.5. Safety Evaluation

DC Injections were done by a physician. After injection, the patient was monitored for two hours for signs of immediate adverse events such as local or systemic allergic reactions, injection site inflammation, or hypersensitivity. Any adverse events that meet Common Terminology Criteria for Adverse Events (CTCAEs) v5.0 were monitored and recorded until seven days after injection.

### 2.6. Laboratory Testing

The Urine Albumin Creatinine Ratio (UACR) was assessed from subjects’ urine. IL6, TNF-α, and IL-10 were measured from serum using sandwich-ELISA kits (Reed Biotech Ltd., Wuhan, China). eGFR was estimated from serum creatinine.

### 2.7. Statistics

Median UACR levels were compared between baseline and weeks 1, 2, 3, and 4 post-intervention. Median of IL6, TNF-α, and IL-10 were compared between baseline and week 4 post-intervention. The Kolmogorov –Smirnov test was used for normality assessment. Normally distributed data were analyzed using the Paired T-test, while non-normally distributed data were analyzed using the Wilcoxon Signed-rank Test. The correlation between UACR and inflammation biomarkers was analyzed using Spearman’s correlation. A multiple linear regression model was constructed to assess the relationship between the change of UACR pre-post treatment (dependent variable) and several covariates, including UACR at Baseline, BMI, cholesterol, HbA1c, eGFR, comorbidity, and medications known to affect albuminuria (ARB, ACE-I, Diuretics, SGLT-2, and GLP-1RA). This analysis aimed to evaluate the effect of these factors on changes in UACR, with all covariates entered simultaneously into the model to control for their potential confounding effects. The achieved statistical power for the linear multiple regression, based on a total sample of 69 subjects, was calculated post-hoc using G*Power version 3.1.9.7 [18]. The protocol of the calculation in G*Power is provided in Supplementary Materials S2. All statistical analyses were performed using IBM SPSS Statistics 22 (IBM, Armonk, NY, USA).

## 3. Results

### 3.1. Subject Characteristics

The study included 69 subjects who met the inclusion and exclusion criteria and completed the study procedures. Even though this sample size meets the minimum samples required for bivariate analysis of UACR change, the posthoc power analysis for multivariate analysis to control confounding factors showed a statistical power of 57.4%, although the ideal is 80%. The average age of subjects was 62 years (range: 39–83 years), with 30 men and 39 women, 36 with microalbuminuria and 33 with macroalbuminuria. Hypertension was the most common comorbidity in 65 (94.2%) subjects. The majority of subjects were overweight (35 subjects, 50.7%). Most subjects had chronic kidney disease (CKD) stage 3b, affecting 23 (33.4%) subjects.

### 3.2. Changes in Albuminuria and eGFR

**Figure 1:**
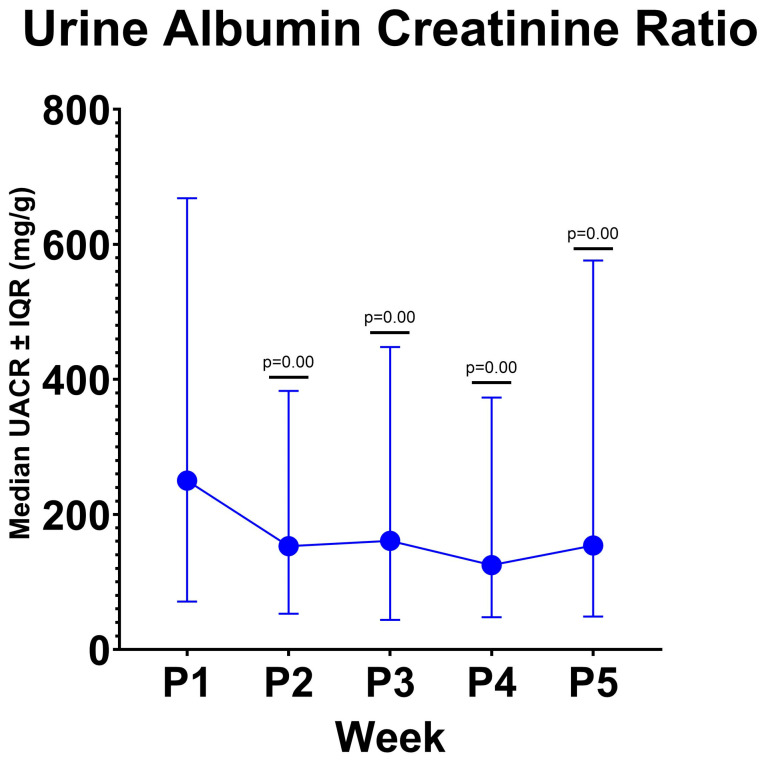
UACR Level by Week. UACR: urine albumin creatinine ratio, *p*:value of statistical significance, IQR: interquartile range.

Each subject had UACR levels measured 5 times: once at baseline (P1) and 4 times post-DC injection (P2–P5) with weekly intervals (Table 1 and Figure 1). The median UACR levels (mg/g) at P1, P2, P3, P4, and P5 were 250, 153, 161, 125, and 164, respectively. The Wilcoxon Signed-ranks Test showed significant reductions in UACR levels from P1 to P2, P3, P4, and P5 (*p* = 0.00) compared to Baseline. Each week’s comparison of UACR is described in Supplementary Materials S3, which shows that UACR levels remained stable from week to week without significant fluctuations after the initial reduction.

To further evaluate kidney function, eGFR at baseline and 4 weeks post-intervention (P5) was compared. No statistically significant change was found. The median eGFR pre-immunotherapy was 60.35 mL/min/1.73 m^2^ (IQR 38.78–86.31), and post-immunotherapy was 56.26 mL/min/1.73 m^2^ (IQR 37.59–90.07 mL/min/1.73 m^2^); no significant changes were detected (*p* = 0.478). These results suggest that immunotherapy did not lead to a noticeable deterioration in kidney function regarding creatinine levels or eGFR following therapy. The eGFR values remained stable, indicating maintained kidney function throughout the duration of the study, which supports the safety profile of the treatment. However, the findings do not demonstrate a significant enhancement in renal function following dendritic cell therapy.

### 3.3. Changes in Inflammatory Biomarkers

**Figure 2:**
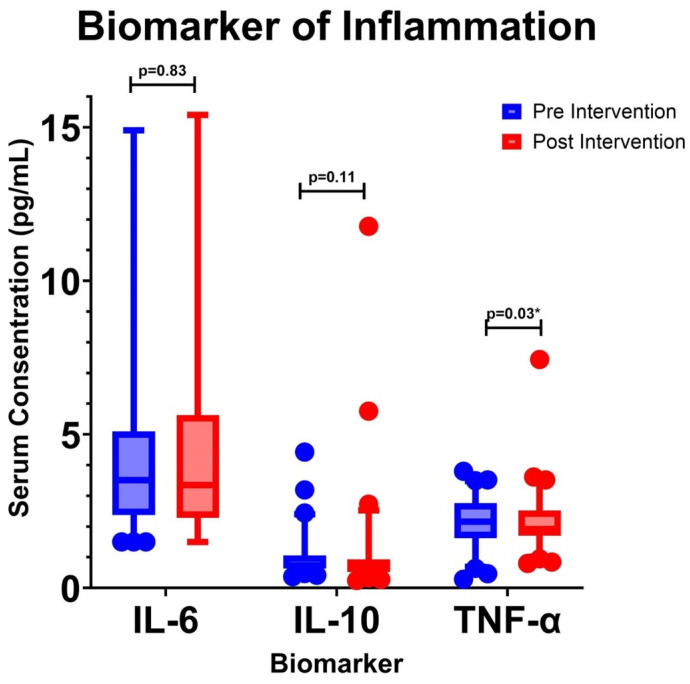
Concentration of Inflammatory Biomarkers. IL-10: Interleukin-10, TNF-α: Tumor Necrosis Factor-α. * *p* < 0.05.

The inflammatory biomarkers measured were IL-10 and TNF-α. IL-10 is a potent anti-inflammatory cytokine, while TNF-α is a pro-inflammatory cytokine. These markers illustrate the anti-inflammatory effects of DC therapy. This study evaluated both biomarkers at P1 and P5 (Table 2 and Figure 2). The Kolmogorov –Smirnov test indicated non-normal distribution. Thus, the Wilcoxon Signed-Rank Test was used. The median IL-6 levels (pg/mL) at P1 and P5 were 3.51 and 3.35, respectively (*p* = 0.83). The median IL-10 levels (pg/mL) at P1 and P5 were 0.74 and 0.63 (*p* = 0.11), while the median TNF-α levels at P1 and P5 were 2.16 and 1.92 (*p* = 0.03). These results indicate a significant decrease in TNF-α but not IL6 and IL-10.

### 3.4. Changes in Ratio of Pro-Inflammatory and Anti-Inflammatory Cytokines

**Figure 3:**
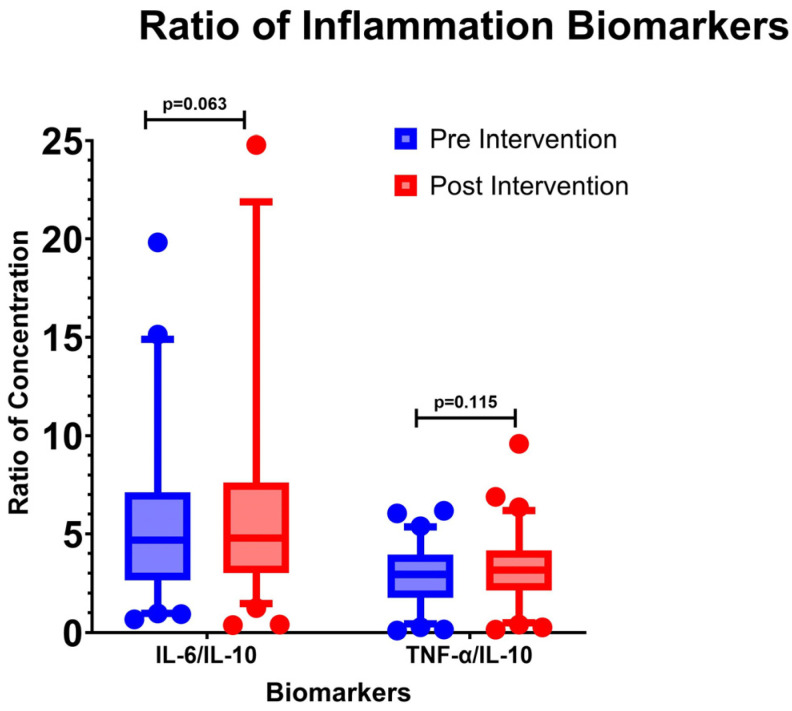
Ratio of pro-inflammatory and anti-inflammatory cytokines in response to treatment. IL-10: Interleukin-10, TNF-α: Tumor Necrosis Factor-α.

The ratio of pro-inflammatory and anti-inflammatory cytokines can reflect the balance of immune responses in the body. In this study, the IL-6/IL-10 and TNF-α/IL-10 ratios were calculated (Table 3 and Figure 3). The IL-6/IL-10 ratio in P1 and P5 was 4.68 and 4.79 (*p* = 0.063), indicating no significant change. Similarly, for the TNF-α/IL-10 ratio, there was no significant difference between P1 (2.94) and P5 (3.16) with a *p*-value of 0.115.

### 3.5. Correlation of UACR and Inflammation Biomarkers

**Figure 4:**
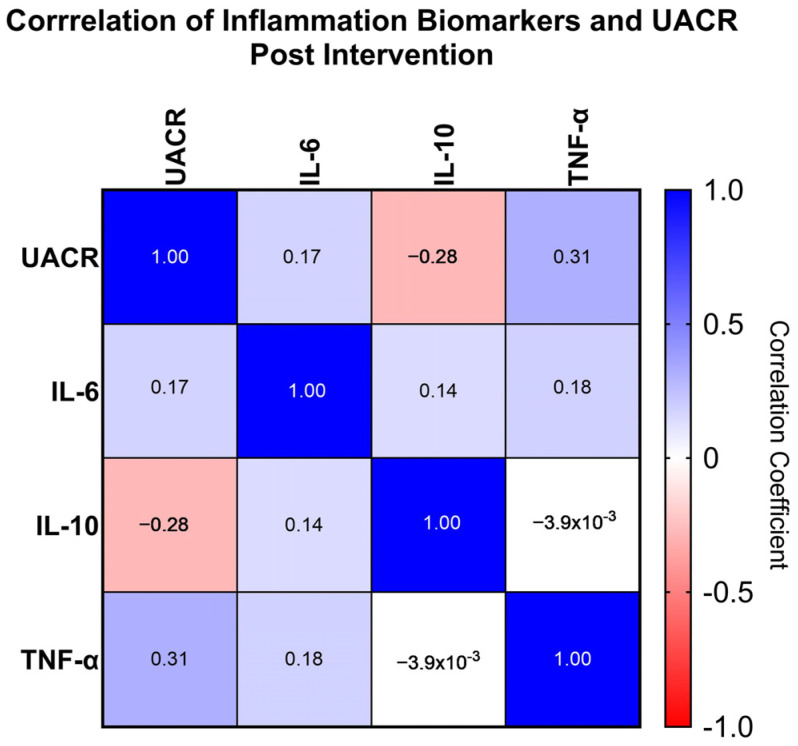
Heatmap of Correlation of UACR and Inflammation Biomarker. IL-10: Interleukin-10, TNF-α: Tumor Necrosis Factor-α.

To examine the interaction between each inflammatory biomarker and UACR post-intervention, a Spearman correlation test was conducted on P5 (Table 4 and Figure 4). Based on the results of this analysis, there was a significant negative correlation between UACR and IL-10, with a correlation coefficient of −0.281 (*p* = 0.019). This indicates that an increase in IL-10 levels tends to be followed by a decrease in UACR. Additionally, there was a significant positive correlation between UACR and TNF-α, with a correlation coefficient of 0.312 (*p* = 0.009). This suggests that an increase in TNF-α levels tends to be followed by an increase in UACR. No significant relationship was found between UACR and IL-6 (correlation coefficient = 0.172, *p* = 0.157). Similarly, the correlations between IL-10 and TNF (correlation coefficient = -0.004, *p* = 0.975), as well as between IL-10 and IL-6 (correlation coefficient = 0.141, *p* = 0.249), were not significant. The correlation between TNF and IL-6 was also not significant (correlation coefficient = 0.179, *p* = 0.141).

### 3.6. Multivariate Analysis

**Figure 5:**
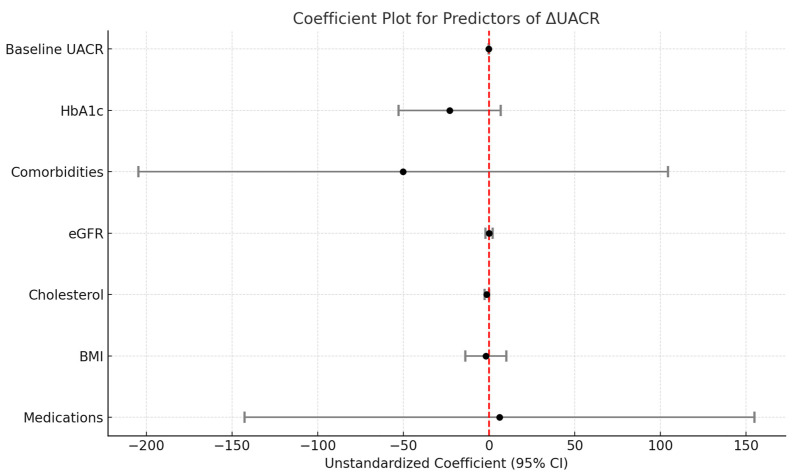
Coefficient Correlation of Multiple Linear Regression of Prediting ΔUACR. eGFR: Estimated Glomerular Filtration Rate, BMI: Body Mass Index, UACR: Urine Albumine Creatinine Ratio, HbA1c: Glycated Hemoglobin.

A multiple linear regression analysis assessed the factors associated with changes in UACR pre and post intervention (ΔUACR) (Table 5 and Figure 5). The model included UACR_Baseline, HbA1c, Medications, BMI, Comorbidities, eGFR, and cholesterol as predictors. The model explained 55.0% of the variance in ΔUACR (*R*^2^ =0.550), with an adjusted *R*^2^ of 0.498, indicating good explanatory power. The overall model was statistically significant (F(7,61) = 10.635, *p*< 0.001), suggesting that the predictors collectively have a significant impact on ΔUACR. Among the predictors, UACR_at baseline (B = −0.159, *p* < 0.001) and cholesterol (B = −1.375, *p* = 0.040) were significantly associated with ΔUACR. Higher baseline UACR levels and cholesterol were associated with a decrease in ΔUACR. The other variables, including HbA1c (B = −22.984, *p* = 0.135), medications (B = 6.154, *p* = 0.936), BMI (B = −1.868, *p* = 0.761), eGFR (B = −0.069, *p* = 0.950), and comorbidities (B = −50.136, *p* = 0.527), did not show significant associations with ΔUACR. Residual analysis revealed a mean residual of 0.00 with a standard deviation of 194.03, indicating substantial unexplained variability in ΔUACR. Standardized residuals ranged from −3.656 to 2.935, suggesting the presence of potential outliers. Despite these outliers, the distribution of residuals was generally well-centered around zero, indicating an overall unbiased fit of the model.

In summary, the results suggest that UACR at baseline and cholesterol are the primary factors influencing changes in UACR, while the other variables showed no significant effects. The model’s unexplained variance suggests that additional factors may need to be considered to enhance the predictive accuracy.

## 4. Discussion

Most of the comorbidities found in the subjects were hypertension. This finding aligns with the study by Soeatmadji et al., which concluded that patients with T2DM in Indonesia predominantly have hypertension as a comorbidity, amounting to 43% [19]. Furthermore, Ismail et al. concluded that individuals in the pre-hypertension and hypertension stages have a higher risk of developing T2DM, with respective RR values of 1.39, 95% CI 1.14–1.69, and RR 1.75, 95% CI 1.43–2.16 [20]. Hypertension and DKD have a complex relationship with interrelated pathophysiological mechanisms. Hypertension not only worsens kidney damage but also arises as a result of renal dysfunction in diabetic patients. In diabetes, increased vascular resistance due to vascular remodeling leads to hypertension. Arteriolar remodeling in DKD raises glomerular pressure, further exacerbating kidney damage and worsening hypertension [21]. This condition is worsened by hyperinsulinemia and fluid overload, which ultimately increase systemic blood pressure [22]. This can explain the findings of this study, where over 90% of diabetic patients with albuminuria—a marker of kidney damage—have hypertension as a comorbid condition.

Overall, there was a significant decrease in UACR from baseline to post-intervention with DC. The median UACR decreased from baseline to the first week, indicating a response to the intervention. A slight increase occurred in the second week, but it decreased again in the third week. Subsequently, a slight increase was observed in the fourth week. However, the median UACR levels from the first to the fourth week after the intervention remained lower than the baseline and were statistically significant. This indicates that DC can reduce UACR in DKD patients for up to four weeks post-intervention.

To date, no studies have examined the effects of reducing UACR in DKD patients following DC administration. Therefore, the reduction in UACR shown in this study can be compared with currently available standard treatments. Telmisartan, an Angiotensin-II Receptor Antagonist (ARB), has been shown to be effective in reducing UACR, with a clinical trial finding that 52% of subjects experienced a 39.6% reduction in UACR from baseline [23]. Enalapril (an ACE inhibitor) can reduce UACR by 65.7% after one year of regular use compared to placebo [24]. GLP-1 Receptor Agonists (GLP-1 RA) have also been shown to significantly reduce UACR compared to placebo, with reductions ranging from 17% to 33% [25]. SGLT2 inhibitors, such as dapagliflozin, can also reduce UACR by 19% to 22% compared to placebo [26]. In this study, the analysis of all subjects revealed that DC administration could reduce UACR by 34.4–50% up to 4 weeks after injection.

IL-10 levels did not change significantly after the intervention. IL-10 is a potent anti-inflammatory and immunosuppressive cytokine produced by macrophages, Th2 cells, DC, B cells, monocytes, neutrophils, eosinophils, and mast cells [27]. T2DM patients have lower IL-10 levels [28]. This is further complicated by immune cell resistance to the anti-inflammatory effects of IL-10 [29]. IL-10 directly reduces glomerular macrophages’ recruitment, activation, and proliferation in vivo. IL-10 significantly reduces macrophage-mediated glomerular injury and improves albuminuria conditions [30].

No significant eGFR change was found after the intervention. Conventional treatment for DKD also found limited effects on eGFR despite long-term treatment [23,24,25,26]. However, some medications are found to protect declining kidney function in diabetic patients. To improve eGFR, a major recovery of kidney structure should occur. Nephron regeneration in chronic kidney disease requires at least twelve weeks, so longitudinal monitoring of eGFR at least every three months should be done [31]. Hence the effect of DC immunotherapy on eGFR remains inconclusive.

This study also found a decrease in UACR levels after DC intervention and a reduction in TNF-α levels. This finding is consistent with previous research showing a positive correlation between TNF-α levels and albuminuria. Several studies have indicated that TNF-α receptors released into circulation can serve as markers of declining renal function in patients with type 2 diabetes mellitus [32,33]. A study found a direct correlation between TNF-α levels and UACR in T2DM patients [34]. A significant increase in TNF-α was also found in DKD patients [35,36]. Multivariate analysis revealed an independent relationship between urinary TNF-α and albuminuria. Furthermore, TNF receptor levels are associated with worsening renal function, even in individuals with normoalbuminuria [37]. Therefore, the decrease in UACR levels following DC administration observed in this study is mediated by a reduction in TNF-α levels.

Pro-inflammatory cytokines play a crucial role in fighting pathogens, while anti-inflammatory cytokines function to prevent tissue damage caused by an excessive immune response. An imbalance in this system can lead to issues: excessive pro-inflammatory conditions can trigger allergies and autoimmune diseases, whereas excessive anti-inflammatory responses can result in immunosuppression [38]. In individuals with DKD, there is often a tendency towards pro-inflammatory overactivation, which can lead to tissue damage [39]. In this study, the results showed no increase in the pro-inflammatory/anti-inflammatory ratio, indicating that the intervention did not alter the immune response balance. Median analysis revealed a significant decrease in TNF-α levels but no change in the TNF-α/IL-10 ratio. This suggests that the reduction in TNF-α was balanced by changes in IL-10, thus posing no risk of immunosuppression.

A Spearman correlation analysis was conducted post-intervention to examine the relationship between the tested parameters. The results indicated that IL-10 and TNF-α after therapy significantly correlated with UACR, while IL-6 did not significantly correlate with UACR or other biomarkers. These findings are consistent with previous studies, where IL-10 was negatively correlated with UACR levels, whereas TNF-α was positively correlated with UACR [40]. Thus, this analysis further supports the conclusion that the reduction in UACR observed post-intervention is mediated by a decrease in TNF-α.

This study has several limitations, including not restricting the use of antihypertensive and antidiabetic medications known to lower UACR, such as RAS inhibitors, GLP-1 Receptor Agonists, and SGLT-2 inhibitors. The short duration of the study (only 4 weeks) limits the evaluation of the long-term impact of the intervention, considering that albuminuria in DKD is progressive. Additionally, the intervention was only performed once, warranting further exploration regarding the optimal frequency and duration to achieve more significant and sustainable therapeutic benefits. Further research is needed to improve medication control, explore the specific effects of DC administration, and determine the most effective dosing and intervention schedule.

Multivariate analysis was conducted to measure the influence of confounding factors on the change of UACR in this study. Cholesterol and baseline UACR contribute significantly to the variance of ΔUACR. Other variables (e.g., BMI, HbA1c, eGFR, complications, and drug use) do not significantly impact the change in UACR, indicating that they may not play a major role in this outcome within the context of this model. Cholesterol is a known predictor of metabolic disorders, and it is known to positively correlate with UACR [41]. Baseline UACR signifies the progression of kidney damage; advanced damage influences the efficacy of this treatment. DC immunotherapy has lower efficacy in people with higher cholesterol and people with more advanced kidney damage. However, due to the limitation of sample size, this study has not reached adequate statistical power (Supplementary Materials S1) to confidently conclude that other variables do not influence UACR. So, follow-up studies involving more subjects should be conducted.

This study was not designed as double-blind due to ethical considerations. In future research, increasing the clinical significance will require patient stratification based on various criteria, such as comorbidity, medication use, degree of kidney damage, vascular remodeling, and glycemic control. A successive clinical trial with a gradually larger cohort that eventually led to a double-blind study design should be conducted.

Another limitation of this study is that dose standardization was not implemented. This is due to a couple of reasons. Firstly, this research did not aim to assess the dose-response relationship; rather, it aimed to determine the effect of dendritic cell (DC) therapy on albuminuria and elucidate its underlying mechanism through inflammatory biomarkers. Additionally, due to the personalized nature of this intervention, the number of autologous DCs administered was based on individual yields, allowing for a tailored therapeutic approach. Nevertheless, standardized dosing is essential for accurately evaluating the dose-response relationship and should be considered in follow-up and future studies. Regardless of the limitations, the significant finding of this study can be the justification for further study.

## 5. Conclusions

This study demonstrates that DC administration can significantly reduce UACR levels in DKD patients for up to four weeks post-intervention compared to baseline, with stabilization of levels in subsequent weeks and deterioration in eGFR. This indicates that the intervention effect was limited to reducing albuminuria without promoting regeneration of glomerular filtration capacity. Additionally, there was a significant reduction in TNF-α levels, supporting the theory that inflammation plays a key role in the pathophysiology of DKD, while IL-10 levels did not change significantly. This suggests a possible anti-inflammatory mechanism of DC administration associated with suppressing the pro-inflammatory cytokine TNF-α rather than promoting the anti-inflammatory cytokine IL-10. Given the low statistical power, further studies with larger samples are recommended to confirm and further investigate the factors associated with treatment response.

## Figures and Tables

**Table 1:** UACR Level by Week.

Visit	UACR Median (25th–75th Percentile) (mg/g)	*p*-Value Hypothesis Test
Baseline (P1)	250 (71–668)	
Week 1 (P2)	153 (53–383)	0.00 *
Week 2 (P3)	161 (44–448)	0.00 *
Week 3 (P4)	125 (48–373)	0.00 *
Week 4 (P5)	164 (49–576)	0.00 *

* *p* < 0.05 (Significant), hypothesis test was done using Wicoxon Sign Rank in comparison to Baseline (P1).

**Table 2:** Serum Concentration of Inflammatory Biomarkers.

	Median (25th–75th Percentile) Pre Intervention/P1 (pg/mL)	Median (25th–75th Percentile) Post Intervention/P5 (pg/mL)	*p*-Value
IL-6	3.51 (2.38–5.06)	3.35 (2.34–5.27)	0.83
IL-10	0.74 (0.63–1.05)	0.63 (0.53–0.91)	0.11
TNF-α	2.16 (1.62–2.74)	1.92 (1.71–2.52)	0.03 *

* *p* < 0.05 (Statistically Significant).

**Table 3:** Ratio of Pro-inflammatory and anti-inflammatory cytokines.

	Median (25th–75th Percentile) Pre Intervention (pg/mL)	Median (25th–75th Percentile) Post Intervention (pg/mL)	*p*-Value
Ratio IL-6/IL-10	4.68 (2.66–7.04)	4.79 (3.03–7.58)	0.063
Ratio TNF-α/IL-10	2.94(1.77–3.85)	3.16(2.21–4.12)	0.115

**Table 4:** Spearman Correlation Inflammation Biomarkers and UACR Post Intervention.

Correlation Coefficient (*p* Value)	IL-6	IL-10	TNF
UACR	0.172 (0.157)	−0.281 (0.019) *	0.312 (0.009) *
IL-6	-	0.141 (0.249)	0.179 (0.141)
IL-10	-	-	−0.004 (0.975)

* *p* < 0.05 (Statistically Significant).

**Table 5:** Multiple Linear Regression Factors Associated with Changes in UACR (ΔUACR).

Predictor ^1^	Unstandardized Coefficient (B)	Std. Error	*p*-Value
(Constant)	508.293	218.750	0.023
Medications	6.154	75.899	0.936
BMI	−1.868	6.116	0.761
Cholesterol	−1.375	0.654	0.040 *
eGFR	−0.069	−0.069	0.950
Comorbidities	−50.136	78.775	0.527
HbA1c	−22.984	15.177	0.135
Baseline UACR	−0.159	0.028	<0.001 *

* *p* < 0.05 (significance); ^1^ Dependent Variable: ΔUACR was calculated from [UACR at Week 4] − [UACR at Baseline].

## Data Availability

The raw data supporting the conclusions of this article will be made available by the authors on request.

## Supplementary Materials

The following supporting information can be downloaded at: https://www.mdpi.com/article/10.3390/cimb46120816/s1, Supplementary Material S1. Sample Size Calculation. Supplementary Material S2. Post-Hoc Power Analysis for Linear Multiple Regression Supplementary Material S3. Comparison of UACR in each week. Reference [42] is cited in Supplementary Materials.
