# Supplementary material for: Open-Label Clinical Trial on the Impact of Autologous Dendritic Cell Therapy on Albuminuria and Inflammatory Biomarkers (Interleukin-6, Interleukin-10, Tumor Necrosis Factor α) in Diabetic Kidney Disease (DKD)
Source: Curr Issues Mol Biol. 2024 Dec 2;46(12):13662–74. doi: 10.3390/cimb46120816 (PMC11727525; doi:10.3390/cimb46120816)
Supplement: Supplementary file 1 [file cimb-46-00816-s001.zip › Supplementary Material S1.pdf]

Supplementary Material S1. Sample Size Calculation

Based on the study by Kashiwagi et al. [1] which measured the average UACR levels in patients with T2DM, a standard deviation (σ) of 497.8 mg/g was found. The sample size was calculated using the sample size formula for comparing means in numerical data for a single population. Using a confidence level of α = 0.05, a power of 0.8, and an expected change in UACR of 150 mg/g, the sample size calculation is as follows:

$$n = \left(\frac{[Z_\alpha + Z_\beta]S}{x_1 - x_2}\right)^2$$

$$n = \left(\frac{[1{,}64 + 0{,}84]497{,}8}{150}\right)^2$$

$$n = 67{,}73$$

With the following parameters:

- n : Sample Size
- S : Standard Deviation
- x1−x2: Effect Size
- Zα : Z-score with alpha 0.05 = 1.64
- Z : Z-score with beta (power) 0.8 = 0.84

The minimum required sample size is 68 subjects to detect a significant change in UACR levels of 150 mg/g.

1. Kashiwagi A, Takahashi H, Ishikawa H, Yoshida S, Kazuta K, Utsuno A, et al. A randomized, double-blind, placebo-controlled study on long-term efficacy and safety of ipragliflozin treatment in patients with type 2 diabetes mellitus and renal impairment: Results of the Long-Term ASP1941 Safety Evaluation in Patients with Type 2 Dia. Diabetes Obes Metab. 2015;17(2):152–60.
