# Supplementary material for: Open-Label Clinical Trial on the Impact of Autologous Dendritic Cell Therapy on Albuminuria and Inflammatory Biomarkers (Interleukin-6, Interleukin-10, Tumor Necrosis Factor α) in Diabetic Kidney Disease (DKD)
Source: Curr Issues Mol Biol. 2024 Dec 2;46(12):13662–74. doi: 10.3390/cimb46120816 (PMC11727525; doi:10.3390/cimb46120816)
Supplement: Supplementary file 1 [file cimb-46-00816-s001.zip › Supplementary Material S2.pdf]

Supplementary Material S2. Post-Hoc Power Analysis for Linear Multiple Regression

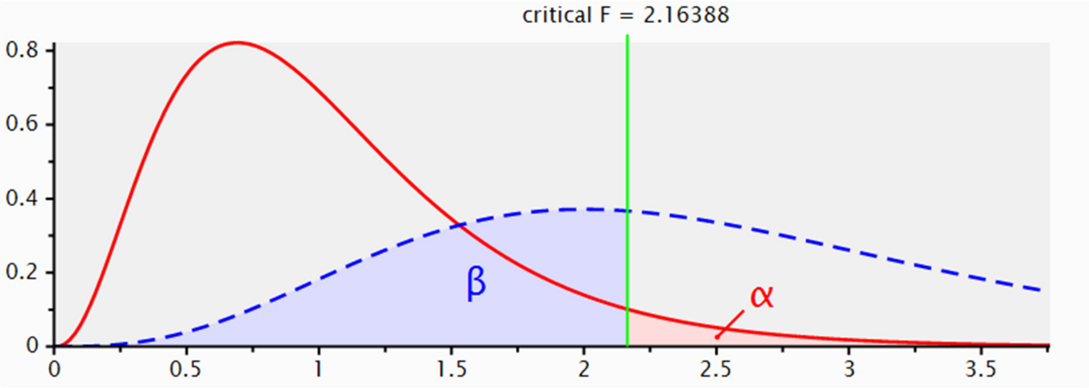

F tests - Linear multiple regression: Fixed model, R^2 deviation from zero

Analysis: Post hoc: Compute achieved power
Input: Effect size f^2=0.15
α err prob=0.05
Total sample size=69
Number of predictors=7
Output: Noncentrality parameter λ =10.3500000
Critical F=2.1638834
Numerator df=7
Denominator df=61
Power (1-β err prob)=0.5739926
