# Supplementary material for: Open-Label Clinical Trial on the Impact of Autologous Dendritic Cell Therapy on Albuminuria and Inflammatory Biomarkers (Interleukin-6, Interleukin-10, Tumor Necrosis Factor α) in Diabetic Kidney Disease (DKD)
Source: Curr Issues Mol Biol. 2024 Dec 2;46(12):13662–74. doi: 10.3390/cimb46120816 (PMC11727525; doi:10.3390/cimb46120816)
Supplement: Supplementary file 1 [file cimb-46-00816-s001.zip › Supplementary Material S3.pdf]

Supplementary Material S3. Comparison of UACR in each week

Visit	UACR Median (*25th-75th Percentile*) (mg/g)[1]	*p-value Hypothesis Test*[1]
Week 1 (P2) vs Week 2 (P3)	153 (53-383) vs 161 (44-448)	0.871
Week 2 (P3) vs Week 3(P4)	161 (44-448) vs 125 (48-373)	0.263
Week 3 (P4) vs Week 4(P5)	125 (48-373) vs 164 (49-576)	0.623

[1]Hypothesis testing was done with Wilcoxon Sign Rank
